# Disentangling help-seeking and giving up: differential human-directed gazing by dogs in a modified unsolvable task paradigm

**DOI:** 10.1007/s10071-021-01595-0

**Published:** 2022-01-12

**Authors:** Annina Hirschi, Alja Mazzini, Stefanie Riemer

**Affiliations:** Companion Animal Behaviour Group, Division of Animal Welfare, Vetsuisse Faculty, University of Bern, 3012 Bern, Switzerland

**Keywords:** Dog *Canis familiaris*, Unsolvable task, Impossible task, Interspecific communication, Referential looking, Gazing

## Abstract

Dogs are renowned for ‘looking back’ at humans when confronted with a problem, but it has been questioned whether this implies help-seeking or giving up. We tested 56 pet dogs from two breed groups (herding dogs and terriers) in a modified unsolvable task paradigm. One reward type (food or toy) was enclosed in a box, while the respective other reward was accessible. With both reward types, human-directed gazing in relation to the box was significantly positively correlated with interaction with the box, as long as an alternative was available. This suggests that both behaviours served to attain the unavailable reward and reflected individual motivation for the inaccessible vs the accessible reward. Furthermore, we varied whether the owner or the experimenter was responsible for handling the rewards. In the owner-responsible group, dogs rarely gazed at the experimenter. In the experimenter-responsible group, dogs preferentially directed box-related gazing (prior to or after looking at or interacting with the box) at the owner. Still, they gazed at the experimenter significantly longer than the owner-responsible group. Conversely, toy-related gazing was directed significantly more at the experimenter. Thus, dogs adjust their gazing behaviour according to the people’s responsibility and their current goal (help-seeking vs play). Gaze duration did not differ between herding dogs and terriers. We conclude that dogs use gazing at humans’ faces as a social problem-solving strategy, but not all gazing can be classified as such. Dogs’ human-directed gazing is influenced by the social relationships with the persons, situational associations, and context (unsolvable problem vs play).

**Supplementary Information:**

The online version contains supplementary material available at 10.1007/s10071-021-01595-0.

## Introduction

Interspecific social-communicative abilities of domestic dogs have been of interest to researchers for several decades, and interest has not waned. Dogs seem to possess unique skills in reading human communicative signals such as pointing or nodding (reviewed in Cavalli et al. 2018; Kaminski and Nitzschner 2013; Miklósi and Soproni 2006). They are also able to flexibly communicate with humans such as to request a reward (Gaunet 2008, 2010; Savalli et al. 2014, 2016; Worsley and O’Hara 2018), and their communicative behaviours are affected by the receiver’s attentive state, the ability to establish eye contact with the person (Brubaker et al. 2019; reviewed in Kaminski and Nitzschner 2013; Marshall-Pescini et al. 2013; Savalli et al. 2016), and prior encouragement (Horn et al. 2012).

In particular, mutual gazing appears to play a key role in dog–human communication (Miklósi et al. 2003; Worsley and O’Hara 2018). In a seminal study, Miklósi et al. (2003) exposed extensively socialised hand-reared young wolves and pet dogs of the same age to two manipulative tasks in which the animals could obtain a food reward. The last trial of each task was blocked, rendering the task unsolvable. In both ‘unsolvable tasks’, dogs were more likely than wolves to look back at the human, the latency to look at the human was shorter, and the duration of looking was higher. The authors concluded that dogs’ ability to gaze at humans’ faces might be a key difference between dogs and their closest extant relatives, grey wolves, and that ‘looking back’ has an important and unique function in dog–human communication (Miklósi et al. 2003).

Since then, variations of the ‘unsolvable task’ paradigm have been used in numerous studies to assess dogs’ cognitive abilities and to tease apart phylogenetic and ontogenetic effects on human-directed gazing in dogs, other canids (reviewed in Cavalli et al. 2018; Mendes et al. 2021), as well as other species (Alterisio et al. 2018; Langbein et al. 2018; Miklósi et al. 2005; Pérez Fraga et al. 2021; Zhang et al. 2021). Several studies demonstrated that wolves show less ‘looking back’ than dogs do (Gácsi et al. 2005; Marshall-Pescini et al. 2017; Miklósi et al. 2003; Udell 2015), while dingoes were found to show intermediate levels of human-directed gazing (Ballard et al. 2021; Johnston et al. 2017).

Cooperative working breeds, such as herding dogs, appear to have a higher propensity for human-directed gazing during unsolvable tasks than those bred for working independently (reviewed in Lazarowski et al. 2020). Furthermore, breeds considered as “primitive” or “wolf-like”, such as the Czechoslovakian wolfdog, tend to gaze less at humans than other breed groups, including herding dogs, hounds, retrievers, and working breeds (Passalacqua et al. 2011; Konno et al. 2016; Maglieri et al. 2019; Sommese et al. 2019). Nonetheless, results have not been consistent across studies. For example, in Lazarowski et al. (2020), human-directed gazing did not differ between dogs from cooperative working breeds (i.e., selected for working in visual contact with humans, including herding dogs and gundogs) and those with no such selective history. In addition, studies reported no difference in gaze duration in Czechoslovakian wolfdogs and German shepherds (Maglieri et al. 2019) (nor between "wolf-like" dogs and molossoids, Passalacqua et al. 2011). Van Poucke et al. (2021) found that herding dogs showed a longer duration of eye contact with the owner during an unsolvable task than both ancient breeds and dogs from solitary hunting breeds (which include Swedish and Norwegian elkhounds, dachshunds, and hunting terriers), who are used in Scandinavian countries to track down game in the forest independently from their owner. Whether the breed histories or individual training experiences contributed to these breed differences could not be differentiated in this study, but likely both play a role.

It is known that individual experiences, including life history (Brubaker et al. 2019; Lazzaroni et al. 2020) and training level, affect human-directed gazing. For example, agility dogs (Marshall-Pescini et al. 2009), search and rescue dogs (Marshall-Pescini et al. 2009), water rescue dogs (D’Aniello et al. 2015), and dogs participating in animal-assisted interventions (Cavalli et al. 2020) display more human-directed gazing than untrained pet dogs. On the other hand, guide dogs (Scandurra et al. 2015) and detection dogs (Lazarowski et al. 2020) show less ‘looking back’ than untrained dogs.

An important factor that needs to be considered when interpreting individual differences in human-directed gazing is persistence, which is often negatively associated with ‘looking back’. Using a solvable task paradigm with pet dogs and socialised wolves, Udell (2015) found that not only did dogs spend more time gazing at humans than wolves, but they also persisted less and were less successful in solving the task than the wolves. Marshall-Pescini et al. (2017) tested highly socialised wolves and similarly raised dogs, pet dogs, and free-ranging dogs in an unsolvable task. The results showed that, independently of species and life experience, lower persistence was associated with shorter latency and longer duration of looking back, with wolves generally being more persistent than dogs. Rather than being a result of domestication, human-directed gazing might indicate animals’ acceptance of humans as social partners, which dogs are predisposed to, but can be achieved in wolves with extensive socialisation (Marshall-Pescini et al. 2017). Thus, Marshall-Pescini et al. (2017) encouraged future research to investigate communicative behaviour more independently from persistence.

Recently, Lazzaroni et al. (2020) questioned whether dogs’ human-directed gazing in an unsolvable task reflects help-seeking behaviour, i.e., an alternative (social) problem-solving strategy, or whether dogs gaze at any salient structure (a person or a non-social object) in the environment as a consequence of giving up. They tested both pet dogs and free-ranging dogs in a modified unsolvable task where either a real (inattentive) unfamiliar human, a ‘dummy’ human, or no person or object was present. Pet dogs were additionally tested with a large human-sized object that bore no resemblance to a human. The duration of looking at the human was higher in pet dogs than in free-ranging dogs (Lazzaroni et al. 2020), as might be expected for communicative behaviour based on different life experiences. Moreover, pet dogs looked significantly longer and more frequently at the real human than at the objects (Lazzaroni et al. 2020), which would be in line with a social function of this behaviour. Nonetheless, Lazzaroni et al. (2020) concluded that ‘looking back’ does not constitute a social strategy. They based this conclusion on the fact that there was no difference in persistence, frequency of looks, and latency to look back between the test populations, and no difference in persistence and latency to first look between the conditions involving a human, an object, or no human (Lazzaroni et al. 2020). They concluded that latency to ‘looking back’ is best explained by individual persistence, while frequency and duration of human-directed gazing depend on stimulus salience and potentially past reinforcement history (Lazzaroni et al. 2020).

Conversely, several studies support the notion that dogs’ human-directed gazing in unsolvable tasks is a social-communicative behaviour (Carballo et al. 2020; Cavalli et al. 2020; Mendes et al. 2021). An indication of help-seeking, as opposed to random gazing at salient objects, could be if dogs direct their gaze differentially at the present persons, depending on their current role in the task or past experiences and reinforcement history. Although an experimenter and the owner or two experimenters were present in many unsolvable task studies, only a few studies have differentiated at whom dogs directed their gazing. It could be expected that when both the owner and a stranger are present during an unsolvable task, dogs gaze more at the owner due to the relationship of dependence between them (Marshall-Pescini et al. 2013) or past reinforcement history (Jakovcevic et al. 2010). Additionally, all other things being equal, a preference for the person handling the rewards could be predicted.

Nonetheless, results regarding the question whether dogs differentiate between an owner or familiar person and an unfamiliar experimenter in their gazing behaviour have been mixed. They seem to be influenced by breed as well as training history, as detailed below. In Maglieri et al. (2019), Czechoslovakian wolfdogs gazed significantly longer at the experimenter (the person who handled the food in the apparatus), German shepherd dogs gazed significantly longer at the owner, and Labrador retrievers exhibited no significant preference for either person (Maglieri et al. 2019). Similarly, in a paradigm where neither person had handled the apparatus or rewards, herding dogs preferred to gaze at and approach their owners, while there was a trend for solitary hunting dogs to seek the proximity of the experimenter (Van Poucke et al. 2021).

In Marshall-Pescini et al. (2009), search and rescue dogs, used to working at a distance from their owner, spent similar amounts of time gazing at the owner and the experimenter, whereas agility dogs focused significantly more on the owner than on the stranger (Marshall-Pescini et al. 2009). In Lazarowski et al. (2020), pet dogs, unlike detection dogs, gazed longer at a familiar person than at a stranger. Also, in Sanford et al. (2018), pet dogs of various breeds spent on average more time gazing at the owner than at the stranger (neither of whom had interacted with the unsolvable task). Conversely, Labrador retrievers showed no preference for looking at either their owner or an unfamiliar experimenter in two studies (D’Aniello et al. 2015; Scandurra et al. 2015). In line with Maglieri et al. (2019), it may be that Labradors, due to their generally high level of sociability, are less selective about whom they direct their gaze at.

To conclude, while different studies have come to different conclusions regarding the function of dogs’ human-directed gazing during unsolvable tasks, the data from previous studies do not allow a clear differentiation between ‘looking back’ due to giving up (i.e., loss of interest in the task) and ‘looking back’ as a social help-seeking strategy, indicating continued motivation for the reward. Therefore, in the current study, we had three objectives:

### Objective 1

First, we aimed to differentiate human-directed gazing due to giving up from gazing as a social problem-solving strategy. To this end, we tested dogs in a novel unsolvable task paradigm in which one reward type (food or toy) was inaccessible in a box, while the respective other reward type was concurrently available. All dogs participated in two conditions, once with a favoured toy enclosed in the box, while a food puzzle, from which the dogs could extract the food with some effort, was freely accessible, and once with the food puzzle filled with food enclosed in the same box, while the toy was available.

Accordingly, when dogs have the opportunity to interact with the alternative reward, the following alternative predictions can be generated:

1. If gazing constitutes a social problem-solving strategy, dogs that are more motivated for the inaccessible reward inside the box than for the available reward will spend more time interacting with the box as well as gazing at people; thus, the duration of interaction with the box and gazing at the people should be positively related.
2. If gazing is a consequence of ‘giving up’, the duration of interaction with the box and gazing at the people should be negatively related.

Furthermore, most previous studies used a test duration of 1 min (reviewed in Cavalli et al. 2018). However, a short testing time might be another reason to account for the negative correlation between interacting with the task and gazing if persistent individuals spend most of this time trying to access the reward (c.f. Miklósi et al. 2003). To address this issue, we used a longer duration of the unsolvable task (3 min). This would increase the likelihood also for highly persistent individuals to gaze at humans (c.f. Marshall-Pescini et al. 2017; Smith and Litchfield 2013). Furthermore, the dogs never experienced a solvable version of the task, as previous successes might increase persistence (Rao et al. 2018).

### Objective 2

Our second objective was to assess the effect of different roles by the present people on dogs’ gazing behaviour. If dogs look at any salient large object nearby, gazing would be distributed equally between the two people present. If dogs primarily form an association with the reward and the person handling the reward in this situation, they would be predicted to gaze longer at the responsible person. If they are primarily influenced by differential previous experiences, or the social relationships, with the present persons, they would be predicted to gaze longer at the owner than at the experimenter.

### Objective 3

Our final objective was to assess the effect of breed group (herding dogs vs terriers) on gazing behaviour and persistence. Since terriers were bred to work independently rather than cooperatively like herding dogs (Dorey et al. 2009; Turcsán et al. 2011), we predicted higher persistence and less human-directed gazing in the terriers compared to the herding dogs.

## Methods

### Subjects

Pet dogs from two breed groups (herding dogs and terriers) between the ages of 1 and 10 years were included in the study (mean age: 5.4 ± 2.7 years; 29 females, of whom 15 neutered; 27 males, of whom 18 neutered; Table S1). Dogs were included if either purebred or crosses between two breeds from within the same target breed group; crossbreeds between different breed groups were excluded. Both experimenters and all included owners were female, since a study indicated that dogs prefer to gaze at women compared to men (Carballo et al. 2020).

A between-subjects design was used. Twenty-nine dogs, including 15 herding dogs and 14 terriers, were included in Group E (experimenter-responsible group). For these dogs, the experimenter performed the unsolvable task procedure, including rendering one of the rewards inaccessible in a box and placing the other reward accessibly on the floor, while the owners were holding their dogs. The dogs of Group E were a subsample of dogs tested in the framework of a play motivation test by A.M. for a different study. The behaviour test included several sequences of play of the dog with the owner (four subtests), free access to a toy while being ignored, as well as two subtests in which the experimenter played with the dog. The unsolvable task was the last interactive test in the sequence. Except for the treatment (identity of the person responsible for rendering the reward inaccessible) and the prior play sequences, the procedures relevant for the current study were identical for Group O, as described below.

Dogs from Group O (owner-responsible group) were recruited to additionally assess the effect of familiarity and/or the dog–human relationship on dogs’ gazing behaviour at the two people during the unsolvable task. In this group, the owners were responsible for handling the rewards and placing them unattainably into the box, while the experimenter (A.H.) was handling the dogs. Twenty-seven dogs were included, including 14 herding dogs and 13 terriers.

The majority of subjects participated in one or several dog sports, including nosework or agility, which have been shown to be associated with opposite effect on dogs’ gazing behaviour. Eleven dogs from Group O and 12 dogs from Group E practised agility or Hoopers agility. Twelve dogs from group O and 13 dogs from group E were trained in a type of nosework (including search and rescue, (man)trailing, and nosework). Of these, nine and eight dogs, respectively, participated in both agility and nosework. Other types of dog sports included obedience, dog dance, disc-dog, and herding. No dog sports were reported for three dogs in Group O and six dogs in group E. For four dogs in group E, these data were not available (Online Resource 1.1).

### Test room

**Fig. 1:**
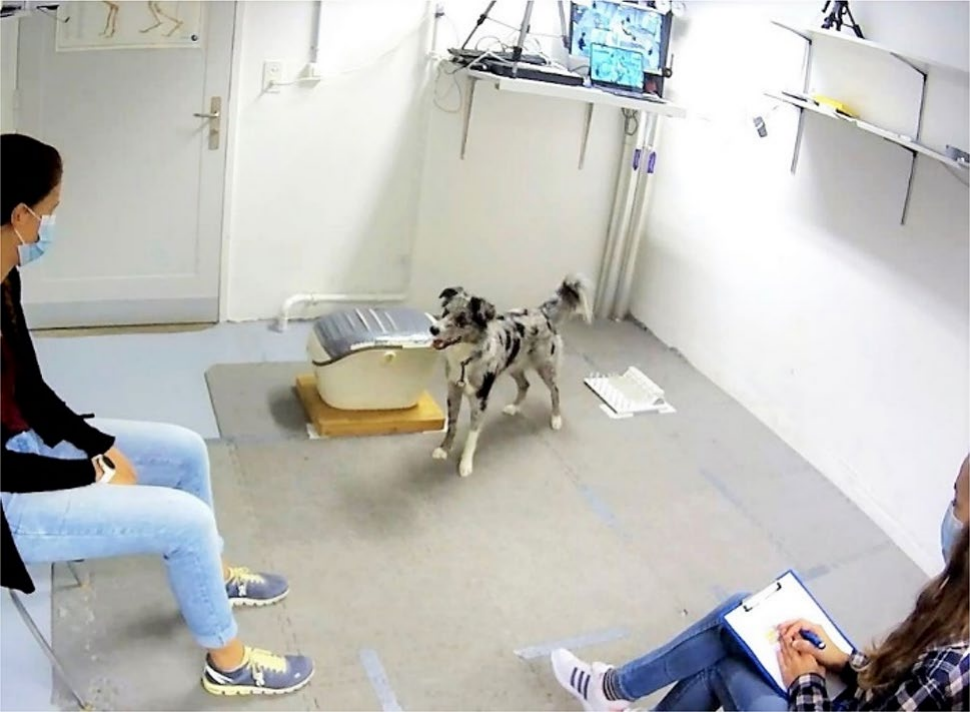
Screenshot of the setup of the test room during the ‘toy in box’ subtest. From this angle, the person responsible for handling the rewards was always seated on the left and the person who handled the dog was seated on the right

Experiments took place in an experimental room (Fig. 1), measuring 5.22 m × 3.36 m, on the campus of the Vetsuisse Faculty, University of Bern (CH). A wooden partition wall divided the room into two parts, so that the effective testing space was 3.60 m × 3.36 m. The room was furnished with two chairs and several shelves on the wall. One of the chairs was placed in front of the wooden partition wall (facing the entrance door); the other chair was placed at a 90° angle against the wall to the left. The person responsible for handling the rewards (Group E: experimenter; Group O: owner) was always seated on the left; the person handling the dog prior to the beginning of the experiments (Group E: owner; Group O: experimenter) was seated on the right, in front of the wooden partition wall (Fig. 1).

The room further contained a water bowl, the unsolvable task box, and the food puzzle. The unsolvable task box was a modified commercial cat carrier (65 × 37 × 31 cm), screwed onto a heavy wooden board. The handle was removed, and the gaps at the top were covered with masking tape to minimise the risk of injury. During all test trials, the box was locked using the secure looking mechanism at the front of the box, which the dogs were unable to open. The food puzzle was a commercially available food puzzle for cats (‘Trixie Cat Activity Fun Board’), a square plastic plate with several pegs, slots, and dents from which food treats could be extracted (c.f. Riemer et al. 2018). To reduce the size of the puzzle to 30 × 30 cm, so that it would fit into the unsolvable task box, an 8 cm-wide part of the puzzle featuring small removable plastic bowls had been cut off. The unsolvable task box and the food puzzle, placed into predetermined positions for the ‘toy in box’ subtest at the front of the room, 40 cm apart, can be seen in Fig. 1. For the ‘food in box’ subtest, the box was placed in the same position, and the toy was placed 40 cm to the right.

Experiments were filmed from four different angles, using Fixed Dome IP cameras. Testing took place between March 2019 and December 2020.

### Procedure

#### Habituation and training phase

After the owner and the dog had entered the room, the dog was unleashed. A habituation phase of 3 min commenced, during which the dog was free to explore the room and the unsolvable task box, which was initially placed against the left side wall. Next, a brief preference test was performed to ensure that the dog was motivated for the toy to be used in the subsequent experiment. The experimenter retrieved a box full of dog toys of various types from the adjacent storage room and asked the owner to select three toys (one ball, one tug toy, and one plush toy) which she thought her dog would like. After removing the remaining toys from the room, the experimenter placed the three selected toys on the floor, approximately 40 cm apart, while the owner was sitting on her chair, holding the dog by the collar or harness. Once the experimenter had returned to her chair, the dog was released and had 30 s to interact with the toys. The toy the dog spent the most time interacting with was subsequently used for the unsolvable task experiment.

After the preference test, the owner was asked to play with the dog with the chosen toy, as they usually would when playing at home, for 1 min. While dogs from Group O then proceeded directly to food puzzle familiarisation, dogs from Group E were tested in further subtests involving toy play due to participating in a different study. These subtests included tug-of-war with the owner, social play without toys with the owner, tug-of-war with the experimenter, and free access to the toy while the owner and experimenter were either present but passive or briefly left the room, as well as playing fetch with both the owner and the experimenter.

All the procedures from then on were identical for the two groups, except that the owner’s and experimenter’s roles of handling the dog and the rewards, respectively, were reversed between the two groups. Before the start of the unsolvable task experiment, all dogs were familiarised with the food puzzle. The puzzle was filled with ten pieces of semi-dry dog food (which is generally highly palatable to dogs), and the dogs had 3 min to extract the food. If needed, the owners were allowed to help and encourage the dog.

#### Unsolvable task procedure

Two subtests were performed directly after each other. In the first subtest, ‘toy in box’, the toy was enclosed in the box, while the food puzzle, filled with 5 pieces of food, was freely available (Fig. 1). In the second subtest, ‘food in box’, the food puzzle, filled with 5 pieces of food, was placed in the box; meanwhile, the toy was freely accessible. Thus, the current study is—to our knowledge—the first study to perform an unsolvable task paradigm with dogs when a different type of reward is concurrently available. By consecutively carrying out the unsolvable test with both reward types, we catered both for dogs with higher toy motivation and for dogs with higher food motivation. The order of tests was fixed for all subjects (first the ‘toy in box’ subtest and then the ‘food in box’ subtest). This fixed order was necessary, because the data from Group E were also used to characterise individual differences in toy motivation for a different study.

In line with Rao et al. (2018), the dogs were never exposed to a ‘solvable’ version of the task, thus preventing possible effects of the previously reinforced manipulative behaviours. The box and the respective alternative reward were placed into predetermined positions at the front of the room, 40 cm apart (see Fig. 1). In Group E (experimenter-responsible group), the owner was sitting on the chair opposite the front of the room where the box and the alternative reward were placed. She held the dog by the collar, harness, or lead, while the experimenter placed the rewards on the floor and into the box, respectively, in full sight of the dog. In Group O (owner-responsible group), roles were reversed. The experimenter was handling the dog, while the owner performed the handling of the rewards and enclosed one of the rewards in the box.

After placing one reward on the floor and enclosing the other one in the box, the person responsible for handling the rewards returned to her chair on the left side of the room, and the person handling the dog released the dog. Each subtest (‘toy in box’ subtest, followed by the ‘food in box’ subtest) lasted 3 min, during which the owner and the experimenter were observing, but did not interact with the dog.

### Coding

Behaviour coding was performed using Solomon Coder (© 2006–2019 by Andràs Péter) at a time resolution of 0.2 s. Durations were coded by the first author. The frequency of gaze alternations was coded by a second, uninvolved coder.

The following variables were coded as durations (full definitions in Table 1): interacting with the box (low effort and high effort; both subtests), interacting with the food or food puzzle (‘toy in box’ subtest), interacting with the toy (‘food in box’ subtest), gazing at the experimenter and gazing at the owner (both subtests). Gazing was only coded when the dog’s gaze appeared to be directed at the person’s face, but not when it was directed at other body parts (c.f. Smith and Litchfield 2013). Finally, drinking was coded as a duration. We subsequently calculated the effective evaluation period for each dog by subtracting the drinking time from the total duration of the subtest.

**Table 1:** Definitions of coded duration variables

Interact food	Interacting actively with food puzzle; the dog’s nose or paw is within 5 cm of food puzzle. Also, interacting with pieces of kibble or chewing	
Interact toy	Interacting actively with the toy; the dog’s nose or paw is within 5 cm of the toy	
Interact box low effort	Interacting actively with low effort with the box; the nose or paw is within 5 cm of the box. Scratch box with one paw or slight nudge. Also coded if the dog circles the box for ≤ 10 s with the nose/paw further away than 5 cm	
Interact box high effort	Interacting actively with high effort with the box. Bite at, paw at, scratch box with two paws, or push box with effort. Intermittent bouts of “low effort” interactions in between “high effort” interactions are coded as “high effort” if they do not last longer than one second	
Look at owner	Any time the dog’s gaze is directed at the owner’s face	Differentiation in the food in box subtest: Box-related gazing: if the dog was interacting with or looking at the box prior to or after gazing at the person Toy-related gazing: if the dog had the toy between the paws or if s/he interacted with or looked at the toy prior to or after gazing at the person
Look at experimenter	Any time the dog’s gaze is directed at the experimenter’s face	
Drink	Dog’s nose is within 5 cm of the water bowl	

Since the toy was concurrently freely available in the ‘food in box’ subtest, the looks at the owner and the experimenter in this subtest were subsequently classified as box-related or toy-related. If the dog had the toy between the paws or if s/he interacted with or looked at the toy prior to or after gazing at the person, this was classified as a toy-related look. If the dog was interacting with or looking at the box prior to or after gazing at the person, this was classified as a box-related look. No such differentiation was made for the ‘toy in box’ subtest, because although the food puzzle was freely available during this subtest, as soon as the five pieces of food were eaten from the puzzle, the puzzle became another “unsolvable task”, and it was less likely that dogs might want to initiate social interaction with one of the persons in relation to the food puzzle than in relation to the toy.

Low effort and high effort interactions with the box were summed up to yield a total duration of interaction with the box. In nine cases, a subtest was terminated up to 3 s early and lasted between 177 and 179 s instead of 180 s. For one dog, the ‘toy in box’ subtest was terminated after 153.6 s, as the dog managed to obtain the toy. For these dogs and dogs that spent part of the time drinking, all durations were extrapolated to 180 s. Analyses are based on extrapolated values.

Gaze alternations, or ‘referential looks’, were coded following the definition after Miklósi et al. (2000), as proposed by Mendes et al. (2021): a gaze alternation was coded when the dog looked at the box and then at a person, or vice versa, within 2 s. Gaze alternations were coded separately as owner-box, box-owner, experimenter-box, and box-experimenter. For the analysis, we used the sums of both types of gaze alternations for the owner and the experimenter, respectively.

#### Reliability

Ten dogs each were coded for reliability by a second, uninvolved coder (one coder: durations of interacting with the box and gaze durations; one coder: gaze alternations). Intra-class correlation coefficients (two-way, random, absolute consistency, single measures) were computed in IBM SPSS Statistics Version 23 (IBM Corporation and its licensors 1989, 2015) and indicated excellent reliability (ICC > 0.9) for all durations and very good reliability (ICC > 0.8) for frequencies (see Tables S2 and S3 for full results).

### Analysis

Statistics were performed in Statistica 6.1. (Statsoft Inc. 1984–2004) or IBM SPSS Statistics Version 23 (IBM Corporation and its licensors 1989, 2015). R version 4.1.0 (The R Foundation for Statistical Computing, 2021) was used to create boxplots. As the data were non-normally distributed, non-parametric statistics were used.

#### Assessment of group differences in food or toy motivation

To assess whether differences in motivation or persistence existed between the owner-responsible and the experimenter-responsible group that might influence the results independently of treatment, we performed Mann–Whitney U tests to compare the two groups in time interacting with the box during both subtests, time interacting with the food puzzle during the ‘toy in box’ subtest, and time interacting with the toy during the ‘food in box’ subtest.

There were no differences between the two treatment groups in persistence in either the ‘toy in box’ subtest or the ‘food in box’ subtest. Moreover, there was no effect of group on the duration of interacting with the food puzzle during the ‘toy in box’ subtest, nor with the toy during the ‘food in box’ subtest (Tables S4 and S5).

#### Effect of face masks on gazing behaviour

From July 2020, the experimenters and the owners wore face masks during the test procedure as a protective measure against COVID-19. As testing of dogs from Group O commenced later than testing of dogs from Group E, all dogs from group O were tested using masks, whereas 17 dogs of Group E were tested without and 12 dogs with masks. To assess possible effects of mask-wearing on dogs’ gazing behaviour, we calculated Mann–Whitney *U* tests comparing gazing behaviour between the dogs from Group E that were tested with vs without masks. The results showed no significant difference in any gazing variables between dogs tested with masks and those tested without masks (Table S6).

#### Changes in gazing behaviour over time (‘food in box’ subtest)

Since our subtests were longer than in most other unsolvable task studies, we aimed to assess possible changes in (box-related) human-directed gazing over time. To this end, we calculated box-related gaze duration for each of the 3 min of the ‘food in box’ subtest separately. A Friedman test was performed to detect possible differences between the three minutes, and a Dunn’s test was used for post hoc pairwise comparisons. For the latter, we report adjusted p values, which are calculated according to the Dunn–Bonferroni approach to correct for multiple comparisons.

Changes over time were not assessed for the ‘toy in box’ subtest, as it was very variable between individuals at what point the food was eaten; therefore, using time only as a predictor would not be as informative. Instead, as described under “Correlations between persistence and human-directed gazing” (below), we analysed gazing behaviour during the ‘toy in box’ subtest both for the whole test period and for the time until the food was consumed.

### Hypothesis testing

#### Correlations between persistence and human-directed gazing

Spearman rank correlation tests were used for correlational analyses. In the ‘toy in box’ subtest, we correlated (1) total interaction time with the box and total time gazing at the two people for the entire subtest (180 s), and (2) the same variables but capping total time when the dog had consumed all the food (individually for each dog), which occurred on average after 81.5 s (SD: 62.8 s).

For the ‘food in box’ subtest, we analysed correlations between interaction time with the box and total time of box-related looks and, separately, total time of toy-related looks.

#### Effect of the responsibility of the owner vs the experimenter

##### Latency to gaze at the owner vs the experimenter

We used Mann–Whitney *U* tests to assess differences between Group O and Group E in the latencies to gaze at the owner and the experimenter, respectively. Within groups, we performed Wilcoxon tests to test whether latencies to first gaze at the two people differed from each other.

If a dog showed no human-directed gazing during a subtest, this was classified as NA and was excluded from the analysis. If a dog gazed only at one of the two people present, we used the maximum duration of the test (300 s) as the latency value for the other person. For the ‘food in box’ subtest, where we differentiated box-related gazing and toy-related gazing, latencies were calculated for box-related gazing only.

##### Proportion of gaze duration directed at the owner

We calculated the proportion of gazing at the owner out of the total time gazing at the two people by dividing the duration of gazing at the owner by the sum of gazing at the owner and the experimenter. Mann–Whitney *U* tests were used to assess whether the two treatment groups differed in proportion of gazing at the owner (1) for the total duration of the ‘toy in box’ subtest, (2) for the total duration of the ‘food in box’ subtest, (3) for the duration of box-related gazing in the ‘food in box’ subtest, and (4) for the duration of toy-related gazing during the ‘food in box’ subtest.

##### Frequency of gaze alternations involving the owner vs the experimenter

The effect of treatment group on the frequency of gaze alternations between the box and the owner and the experimenter, respectively, was also analysed with Mann Whitney *U* tests.

#### Effect of breed group

Data from Group O and Group E were combined to assess possible effects of the breed group, since the treatment groups were balanced for breed group. The effect of breed group on total time interacting with the box (both subtests), total time looking at the two people (both subtests), and total time interacting with the food or food puzzle (‘toy in box’ subtest) and the toy (‘food in box’ subtest) was analysed using Mann–Whitney U tests.

### Correction for multiple testing

Sequential Bonferroni was applied separately for each of the six families (Voelkl 2019) of tests (1—correlations of persistence with gazing; 2—group differences in latency to gaze at the owner vs the experimenter; 3—within-group differences in latency to gaze at the owner vs the experimenter; 4—group differences in the proportion of gazing at the owner; 5—group differences in gaze alternations; 6—differences between the breed groups). With two exceptions, significant results remained significant after correction. In the Results, we only mention the correction when the significance of results changed when using the corrected alpha level. The full results, including the corrected alpha levels, are presented in Table S7.

## Results

### Changes in gazing behaviour over time (‘food in box’ subtest)

A Friedman test comparing the total duration of human-directed gazing in the full sample of 56 dogs between the 3 min of the ‘food in box’ subtest was significant (test statistic = 15.57, *p* < 0.001). Post hoc Dunn tests revealed a significant difference between minute 1 and minute 2 (test statistic = -0.571, adjusted *p* = 0.007), but not between minutes 1 and 3 (test statistic = -0.366, adjusted *p* = 0.158), nor between minutes 2 and 3 (test statistic = 0.205, adjusted *p* = 0.832).

**Fig. 2:**
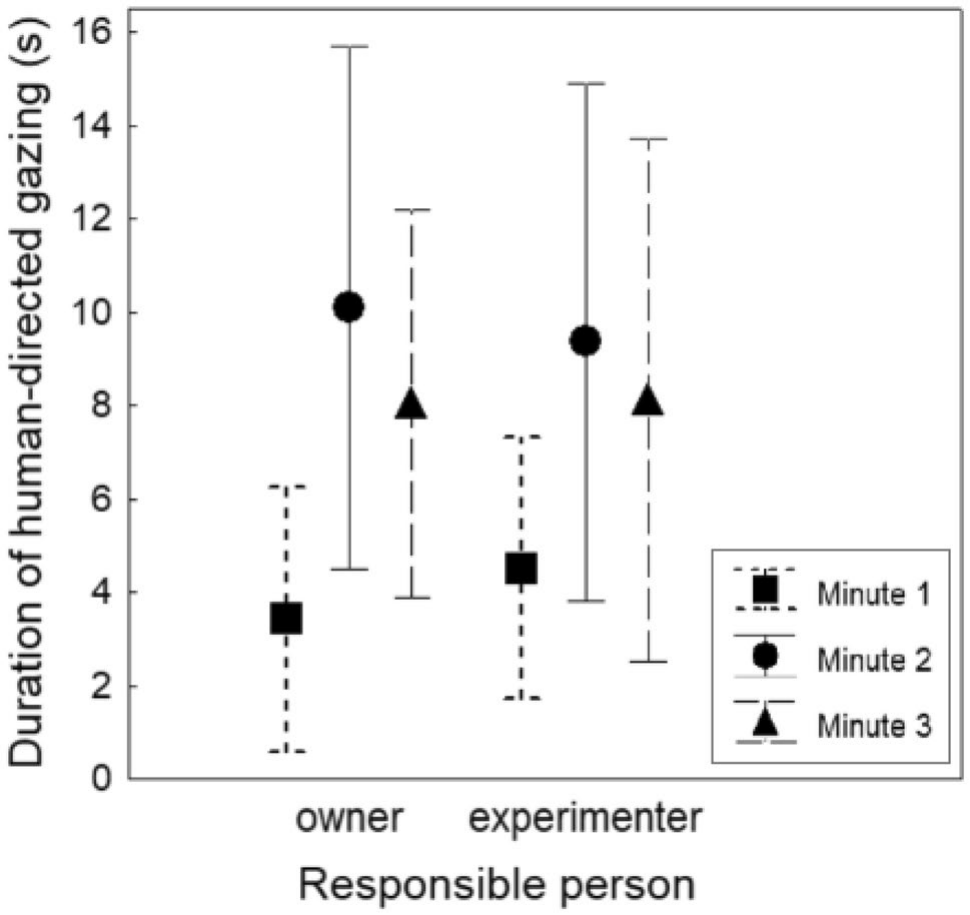
Mean and interquartile range of human-directed gaze duration during the first, second, and third minute of the ‘food in box’ subtest, presented separately for the owner-responsible and the experimenter-responsible group

As all medians were zero, these are not shown graphically. Instead, Fig. 2 shows the means and confidence intervals of human-directed gaze duration during minutes 1, 2, and 3 of the ‘food in box’ subtest, separated by treatment group.

### Correlations between persistence and human-directed gazing

**Fig. 3:**
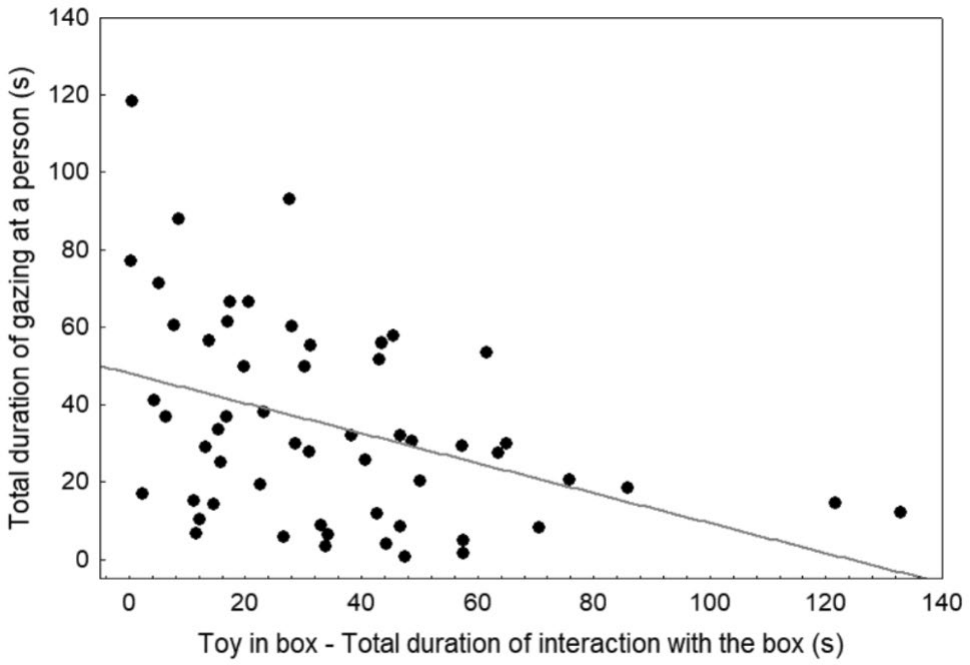
Association between duration of interaction with the box (s) and human-directed gazing (s) during the entire duration (3 min) of the ‘toy in box’ subtest

**Fig. 4:**
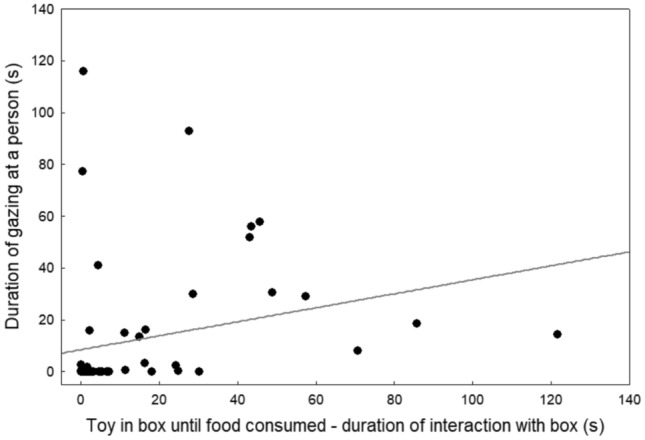
Association between duration of interaction with the box (s) and human-directed gazing (s) during the ‘toy in box’ subtest when food was still available

When analysing all three minutes of the ‘toy in box’ subtest, there was a significant negative correlation between interaction time with the box and duration of gazing at people (*r*_S_ = − 0.42, *p* = 0.001) (Fig. 3). On the contrary, when analysing the subtest only until the food had been consumed, there was a significant positive correlation between interacting with the box and gazing at people (*r*_S_ = 0.51, *p* < 0.0001) (Fig. 4).

**Fig. 5:**
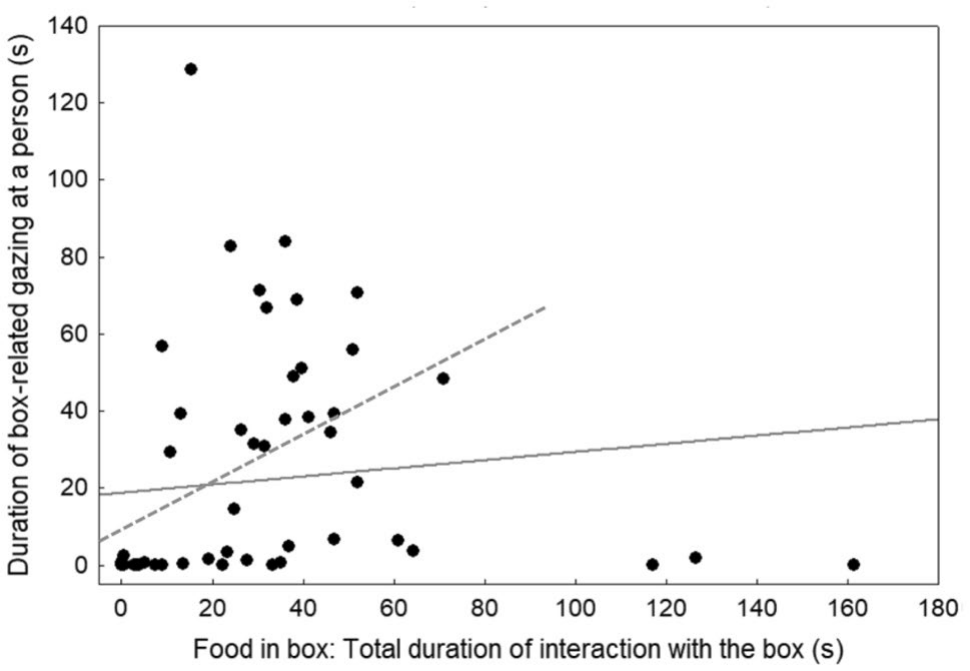
Association between total duration of interaction with the box (s) and duration of box-related gazing (s) at a person (owner and experimenter combined) in the ‘food in box’ subtest. The solid grey line indicates the regression line with the three highly persistent outliers included. The dashed line indicates the regression line with the three outliers excluded

For the ‘food in box’ subtest, where a toy was available throughout, persistence in interacting with the box and total human-directed gazing (including box-related and toy-related gazing) were likewise positively correlated (*r*_S_ = 0.29, *p* = 0.029). However, the correlation coefficient was low, and the relationship was not significant following sequential Bonferroni correction. When analysing only box-related gazing, however, this was highly positively correlated with persistence (*r*_S_ = 0.55, *p* < 0.0001) (Fig. 5).

All data points were included in the statistical analysis, but for visualisation purposes, we present a regression line both with (solid line) and without the three most persistent dogs (dashed line) in Fig. 5. These dogs can be considered outliers, as shown in the boxplot (Fig. S1).

There was a negative association between toy-related gazing and persistence (*r*_S_ = − 0.28, *p* = 0.0334).

### Effect of the responsibility of the owner vs the experimenter

#### Latency to gaze at the owner vs the experimenter

##### ‘Toy in box’ subtest

In the ‘toy in box’ subtest, there was no significant difference in latency to gaze at the owner between Group O (median = 56.9 s, IQR 41.8–95) and Group E (median = 48 s, IQR 16.6–77.6) (Mann–Whitney *U* test, *U* = 293.5, *p* = 0.16). In contrast, there was a highly significant difference in latency to gaze at the experimenter (Group O: median = 89.9 s, IQR 46.6–117.6; Group E: median = 46.4 s, IQR 17.8–70.4) (Mann–Whitney *U* test, *U* = 146, *p* < 0.0001).

In Group O, the dogs gazed significantly sooner at the owner (median = 56.9 s, IQR 41.8–95) than at the experimenter (median = 89.9 s, IQR 46.6–117.6) (Wilcoxon, *N* = 26, *Z* = 2.96, *p* = 0.003). In Group E, latencies to gaze at the two people (owner: median = 48 s, IQR 16.6–77.6; experimenter: median = 46.4 s, IQR 17.8–70.4) did not differ significantly (Wilcoxon, *N* = 29, *Z* = 0.23, *p* = 0.82).

##### ‘Food in box’ subtest

In the ‘food in box’ subtest, the two groups did not differ in their latencies to gaze either at the owner (Mann–Whitney *U* test, *U* = 149, *p* = 0.14) or the experimenter (Mann–Whitney *U* test, *U* = 172, *p* = 0.40). Within Group O, the latency to gaze at the owner (median = 66.4 s, IQR 26.6–84.2 s) tended to be shorter than the latency to gaze at the experimenter (median = 81.6 s, IQR 63.2–147) (Wilcoxon, *N* = 17, *Z* = 2.34, *p* = 0.019; not significant after correction for multiple testing). In Group E, although the median latency to gaze at the owner (median = 73.6 s, IQR 35.2–259.9) was longer than the latency to gaze at the experimenter (median = 43.7 s, IQR 26–234.1), this difference was not significant (Wilcoxon, *N* = 24, *Z* = 0.31, *p* = 0.75).

#### Proportion of gaze duration directed at the owner vs the experimenter

**Fig. 6:**
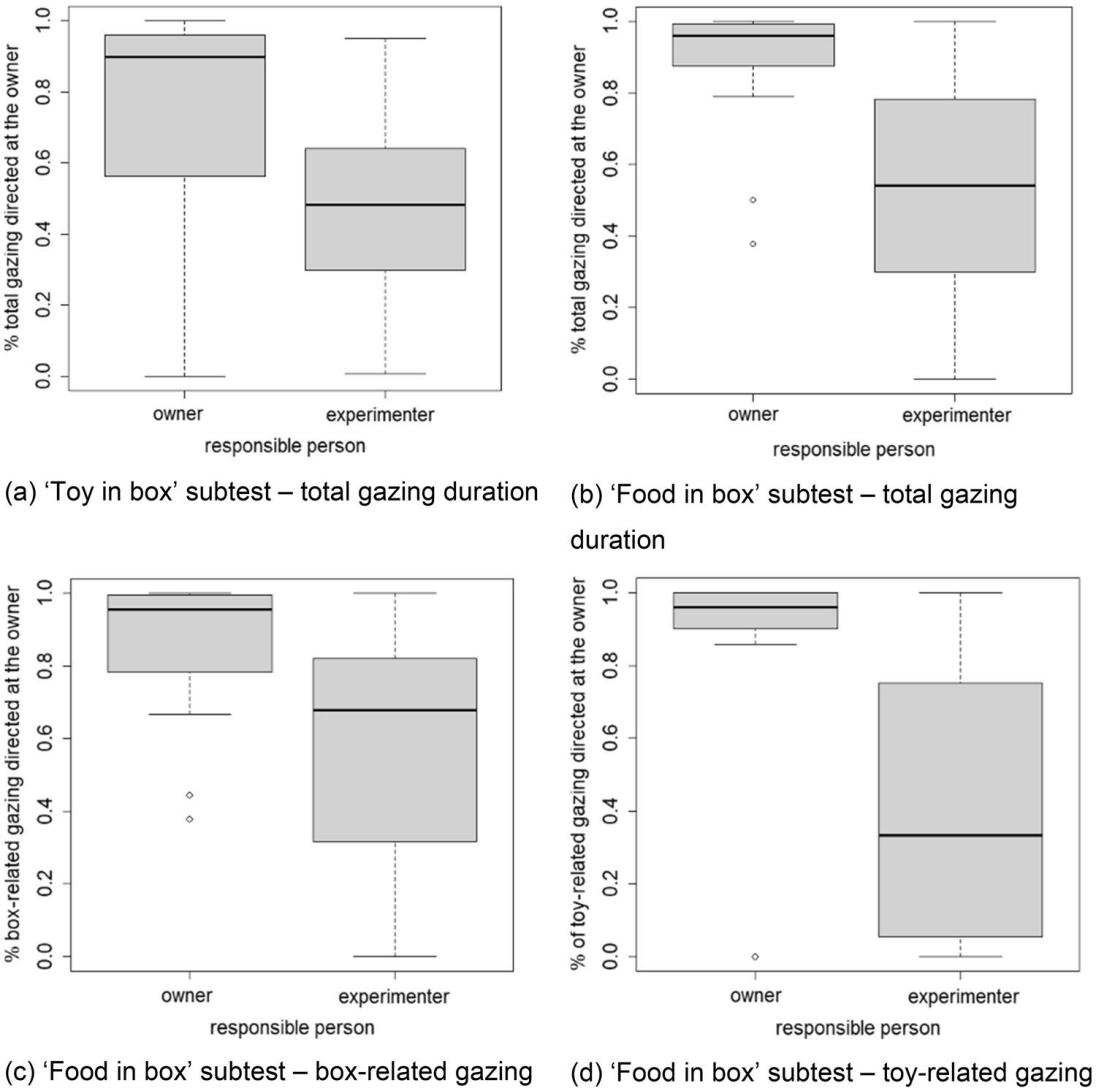
Proportion of gazing directed at the owner in the owner-responsible and the experimenter-responsible group during the ‘toy in box’ subtest (**a**) and the ‘food in box’ subtest [total gazing duration (**b**); box-related gazing (**c**); and toy-related gazing (**d**)]

The proportion of looking at the owner differed significantly between treatment groups in all contexts tested (Tables 2 and 3): total gaze duration during the ‘toy in box’ subtest (Group O: median proportion of gazing directed at the owner = 0.89; Group E: median = 0.48) (Fig. 6a); total gaze duration during the ‘food in box’ subtest (Group O: median = 0.96; Group E: median = 0.54) (Fig. 6b), as well as when splitting box-related gazing (Group O: median = 0.92; Group E: median = 0.70) (Fig. 6c) and toy-related gazing (Group O: median = 0.96; Group E: median = 0.30) (Fig. 6d) during the ‘food in box’ subtest.

**Table 2:** Results of Mann–Whitney *U* tests comparing the proportion of gazing at the owner (from the total time gazing at the two people present) between the two treatment groups

Subtest	Dependent variable	*U*	*p*
Toy in box	Proportion of total gaze duration directed at the owner	143	0.00005
Food in box	Proportion of total gaze duration directed at the owner	90	0.00001
Food in box	Proportion of box-related gaze duration directed at the owner	95.5	0.035
Food in box	Proportion of total toy-related gaze duration directed at the owner	67.5	0.00097

**Table 3:** Median and interquartile range of the proportion of total gazing duration directed at the owner in the experimenter-responsible group (Group E) and the owner-responsible group (Group O)

Subtest	Dependent variable	Group O valid *N*	Group O median proportion of gazing directed at the owner ± IQR	Group E valid *N*	Group E median proportion of gazing directed at the owner ± IQR
Toy in box	Proportion of total gazing duration directed at the owner	27	0.90 (0.56; 0.97)	29	0.48 (0.29; 0.64)
Food in box	Proportion of total gazing duration directed at the owner	23	0.96 (0.56; 0.99)	28	0.54 (0.30; 0.78)
Food in box	Proportion of box-related gazing duration directed at the owner	18	0.92 (0.76; 0.99)	18	0.71 (0.33; 0.94)
Food in box	Proportion of total toy-related gazing duration directed at the owner	19	0.96 (0.76; 1.00)	19	0.31 (0.0; 0.75)

Valid *N* indicates the number of dogs that showed any gazing at all during a given context; dogs that did not gaze at either person were excluded from the respective analyses

#### Frequency of gaze alternations involving the owner vs the experimenter

Highly significant differences between the owner-responsible and the experimenter-responsible group were found in the frequency of gaze alternations directed at the experimenter, both in the ‘toy in box’ and in the ‘food in box’ subtest (Table 4). Conversely, the number of gaze alternations directed at the owner did not differ significantly between the treatment groups (Table 4). In the ‘toy in box’ subtest, the median number of gaze alternations in the experimenter-responsible group was 4 involving the owner (IQR 2–10) as well as 4 involving the experimenter (IQR 2–7). In the owner-responsible group, the corresponding numbers were 5 gaze alternations involving the owner (IQR 2–9) and only 1 gaze alternation involving the experimenter (IQR 0–2). During the ‘food in box’ subtest, the experimenter-responsible group showed a median of 1 gaze alternation directed at the owner (IQR 0–5) and the same number directed at the experimenter (IQR 0–3). In the owner-responsible group, a median number of 2 (IQR 0–5) gaze alternations involved the owner, while for the experimenter, the median number of gaze alternations was 0 (IQR 0–1).

**Table 4:** Results of Mann–Whitney *U* tests comparing the frequency of gaze alternations involving the owner and the experimenter, respectively, between the owner-responsible and the experimenter-responsible group

Subtest	Dependent variable	*U*	*p*
Toy in box	Number of gaze alternations involving the owner	370.5	0.73
Toy in box	Number of gaze alternations involving the experimenter	134	0.00002
Food in box	Number of gaze alternations involving the owner	387	0.94
Food in box	Number of gaze alternations involving the experimenter	208.5	0.0027

### Effect of breed group

All statistical results are shown in Table 5. During the ‘toy in box’ subtest, interaction time with the box was significantly longer in terriers than in herding dogs. No such differences were observed in the ‘food in box’ subtest. The breeds also did not differ in the duration of interaction with the food puzzle nor with the toy when available. Moreover, gazing behaviour (both total gazing duration and proportion of gazes directed at the owner) did not differ between the breed groups in any subtest.

**Table 5:** Results of Mann–Whitney *U* tests analysing effects of breed group on total interaction time with the box during both subtests, duration of interaction with the food or food puzzle (‘toy in box’ subtest), duration of interaction with the toy (‘food in box’ subtest), time gazing at a person (owner and experimenter combined), and proportion of gazing at the owner in both subtests

Subtest	Dependent variable	*U*	*p*
Toy in box	Total time interacting with box	221	0.005
Food in box	Total time interacting with box	374.5	0.780
Toy in box	Total time interacting with food (puzzle)	350	0.496
Food in box	Total time interacting with toy	342	0.417
Toy in box	Total time gazing at a person	304	0.151
Food in box	Total time gazing at a person	371.5	0.743
Toy in box	Proportion of total gaze duration directed at the owner	381	0.863
Food in box	Proportion of total gaze duration directed at the owner	314	0.850

## Discussion

Our study provides several lines of evidence for the communicative nature of human-directed gazing during an unsolvable task in pet dogs. First, we were able to control for motivation by allowing dogs access to a different reward type (food or toy), while the respective other reward type was enclosed in the unsolvable task box. When an alternative reward type was available, duration of interaction with the box and (box-related) gazing were highly positively correlated, indicating that ‘looking back’ constitutes an alternative, social, problem-solving strategy under these circumstances. This was the case both during the ‘toy in box’ subtest as long as food was available and during the ‘food in box’ subtest, where a toy was accessible throughout. Thus, when the reward the dogs were more motivated to have was enclosed in the box, they spent more time trying to access it as well as more time gazing at the people (in relation to the box enclosing the reward).

Second, the dogs adjusted gazing behaviour depending on who (owner or experimenter) was responsible for handling the rewards. When the owner was responsible, the dogs rarely looked at the experimenter at all. When the experimenter was responsible, approximately one-third of box-related gazes were directed at the experimenter and two-thirds at the owner. Thus, while the dogs preferred to gaze at the owner when faced with an unsolvable problem, they demonstrated sensitivity to the people’s roles. This is also reflected to some extent in latencies to gaze at the owner vs the experimenter. During the ‘toy in box’ subtest, in Group O, latency to gaze at the owner was significantly shorter than latency to gaze at the experimenter, while no significant difference was seen in Group E. During the ‘food in box’ subtest, the differences were not significant. Thus, while we see some group differences in latencies, as in previous studies, latencies appear to be less sensitive to treatments or test populations than durations (e.g., Lazzaroni et al. 2020).

Intriguingly, in contrast to box-related gazing, the dogs in the experimenter-responsible group spent more time directing toy-related gazing at the experimenter than at the owner. Hence, gaze direction cannot solely be explained by direct associations with the person handling the reward. Furthermore, since the dogs differentiated between the two people depending on who had handled the rewards, this rules out that differences in visual salience between the people were the determining factor for gazing.

Thirdly, gaze alternations are considered as an indicator that human-directed gazing is referential (Gaunet 2010; Gaunet and Deputte 2011; Marshall-Pescini et al. 2013; Miklósi et al. 2000; Pérez Fraga et al. 2021; Smith and Litchfield 2013). In the current study, as in previous studies, dogs performed gaze alternations, and the direction of gaze alternations was affected by the responsibility of the two persons present. Dogs alternated their gazes between the box and the owner equally often in the owner-responsible and the experimenter-responsible group. However, while dogs from the owner-responsible group rarely directed referential looks at the experimenter, in the experimenter-responsible group, gaze alternations involved the experimenter and the owner at similar rates. Notably, we only considered gazing at the people’s faces, but not other body parts, when coding gazing duration and frequency of gaze alternations. This minimises the chance that random looking around was included in our measure of human-directed gazing.

Still, as already pointed out by previous authors (Brubaker et al. 2019; Smith and Litchfield 2013), not all ‘looking back’ is referential. Thus, although gazing in the ‘toy in box’ subtest was positively associated with persistence as long as food was still available, when analysing all 3 min of the subtest (dogs had consumed all food on average after 81 s), the relationship between box-related gazing and interacting with the box was negative, as found in previous studies (Marshall-Pescini et al. 2017; Udell 2015). When an alternative is available, both interaction with the task and gazing appear to reflect motivation to obtain the inaccessible reward, with the preferred reward type differing between subjects (c.f. Bremhorst et al. 2018, 2021). Conversely, when no alternative is available, ‘looking back’ probably partly reflects help-seeking and partly giving up, leading to the observed negative correlations in this and other studies (Marshall-Pescini et al. 2017; Udell 2015).

An interesting finding is the differential toy-related and box-related gazing behaviour during the ‘food in box’ subtest. When the owner was responsible for handling the rewards, the dogs rarely directed box-related (8%) or toy-related gazes (4%) at the experimenter. Even dogs in the experimenter-responsible group had a strong tendency to gaze at their owner in relation to the unsolvable task: 71% of box-directed gazing was directed at the owner. In contrast, toy-related gazing in Group E was directed predominantly (69%) at the experimenter, even though she was unfamiliar to the dogs prior to the test, and the owner had spent more time playing with them (during four subtests) than the experimenter (during two subtests). While the prior play history with the experimenter in Group E could potentially explain differences between Groups O and E in experimenter-directed gazing, it cannot explain the relative preference for toy-related gazing at the experimenter compared to the owner in Group E, as the owner had played with the dogs in twice as many subtests as the experimenter.

The results suggest that it is not simple reinforcement history that caused dogs to predominantly gaze at the owner in relation to the unsolvable problem. If so, we would have expected similar results for box-related and toy-related gazing. Thus, neither short-term experiences (during the behaviour test) nor long-term experiences (during everyday life) could account for the differential results regarding box-related and toy-related gazing. Dogs seemed to preferably gaze towards the owner when in need of help, but to direct play invitations more to the person who had last handled their reward, despite the very different lifetime reinforcement histories with the two people present.

Turning to the caregiver in “times of need” may reflect the attachment relationship, as seen in social referencing in relation to a potentially threatening novel object (Merola et al. 2012). The higher duration of toy-related gazing at the experimenter when she was responsible for handling the rewards would also be consistent with a characteristic of attachment, namely the secure base effect. Since the owner was present throughout the test, dogs might feel safe to invite the experimenter to play, and here an association of the reward and the person who handled it, as suggested by Lazzaroni et al. (2020), might come into play.

While past training history has been found to affect dogs’ gazing behaviour, the observed group differences in the current study appear to be best be explained by the treatment (identity of the person responsible for handling the rewards), since similar numbers of dogs in Group E and Group O participated in agility and nosework, which in previous studies were found to be associated with differential propensity to gaze at a familiar vs an unfamiliar person (Lazarowski et al. 2020; Marshall-Pescini et al. 2009).

To conclude, our study design using two reward types concurrently enables some differentiation (albeit not perfect) of referential gazing (primarily when an alternative is available) and non-referential gazing (presumably more common when there is no alternative). The data provide a strong indication that dogs gaze at humans’ faces as a communicative act in an apparent request for help, but not all looking can be classified as referential. This study thus expands on previous research which demonstrated evidence of the communicative nature of ‘looking back’ in dogs. As reviewed in Mendes et al. (2021), communicative intent can be inferred from behavioural differences depending on the presence or absence (or the attentive state) of an audience, successive gaze alternations, attention-getting behaviours (e.g., barking), persistence, and elaboration when the recipient fails to respond. All of these behaviours have been observed in dogs in relation to an unattainable food reward (e.g., Gaunet 2008; Gaunet and Deputte 2011; Marshall-Pescini et al. 2009, 2013; Miklósi et al. 2000; Savalli et al. 2014; reviewed in Mendes et al. 2021).

### Methodological considerations

The wearing of face masks by the present people did not significantly affect dogs’ communicative behaviours in the current study. A previous study has shown that dogs were able to match human emotional expressions even when only the upper or only the lower half of a person’s face was visible (Müller et al. 2015). Thus, current data indicate that dogs can adapt well to humans’ mask-wearing, as has become common since the start of the COVID-19 pandemic.

The analysis of changes in gaze duration over time in the ‘food in box’ subtest demonstrated an increase in gazing during minute 2 compared to minute 1, as would be expected if dogs initially try to solve the task on their own and, when unsuccessful, turn to the human(s) at some point (c.f. Marshall-Pescini et al. 2017; Miklósi et al. 2003; Smith and Litchfield 2013). This suggests that carrying out unsolvable tasks for at least 2 min would indeed be favourable to avoid confounds due to individuals’ persistence, and results might be even clearer when the test is three minutes long, as there was only a slight decrease in gaze duration from minute 2 to minute 3.

It could be questioned whether our findings were affected by the fact that, unlike in most unsolvable task studies (but see Rao et al., 2018), the dogs in our study never experienced a solvable version of the task. However, a number of studies demonstrated communicative attempts by dogs in relation to unavailable rewards that were out of reach from the start, such as on a shelf (e.g., Miklósi et al. 2000; Savalli et al. 2014, 2016). Thus, based on these previous findings and in light of our results, we are confident that our modified unsolvable task was suitable to induce communicative behaviours in dogs.

It is a possibility that animals would be more persistent after previous successful trials than when the task is unsolvable from the start (indeed, possible reinforcement of specific operant behaviours during the solvable task has been suggested as a drawback of the inclusion of solvable trials preceding the unsolvable one, Rao et al. 2018). However, recently, Johnston et al. (2021) specifically tested how dogs’ behaviour towards an unsolvable puzzle was affected by having prior experience with solving the puzzle task as opposed to having observed a demonstration by the experimenter only. There was no significant difference in the duration of interaction with the unsolvable puzzle, nor in the duration of looking back at the owner between dogs that had experience with solving the puzzle and those that did not (Johnston et al. 2021). In the current study, the dogs spent a significant proportion of time interacting with the box as well as gazing at people, demonstrating that they were sufficiently motivated to try to access the unavailable reward as well as to employ communicative strategies to achieve this goal. For this reason, we do not feel that the fact that there was no solvable trial significantly impacted the relevance of our results.

### Breed group effects

Contrary to the predictions, no significant differences between herding dogs and terriers in gazing behaviour were found. Nor did the breeds differ in persistence during the ‘food in box’ subtest, interest in the food puzzle, interaction with the available toy, and either box-related or toy-related gazing. The only difference was persistence in the ‘toy in box’ subtest, where terriers spent significantly more time interacting with the box.

One reason for the lack of difference in gazing between the two breed groups might lie in the fact that most of the participating dogs were well trained and took part in dog sports with their owners. Several types of training are associated with increased human-directed gazing (Marshall-Pescini et al. 2009; D’Aniello et al. 2015), so perhaps training effects trumped heritable differences in propensity for making eye contact in this case. Furthermore, most dogs had been trained to take up eye contact by their owners. It is known that this is a behaviour that is easily trained through positive reinforcement (Bentosela et al. 2008). However, breeds were found to differ in the extinction of this behaviour when unrewarded, with Labrador retrievers maintaining gazing for longer than German shepherds and poodles (Jakovcevic et al. 2010). In addition, in Sundman et al. (2018), German shepherds dogs exhibited less human-directed gazing than Labradors, which was suggested to imply higher cooperativeness by the retrievers. Even so, our sample of 29 herding dogs only included two German shepherds but, besides other breeds, 11 Border collies, which are generally regarded as highly cooperative. Thus, it is unlikely that the breed selection within the breed groups can explain the lack of a difference between the terrier and the herding dog group. It is, however, a possibility that the fact that an alternative reward was available attenuated possible differences in propensity for human-directed gazing between the breed groups.

Finally, terriers were more persistent than herding dogs in the ‘toy in box’ subtest, but there were no breed differences in the ‘food in box’ subtest. Since gazing did not differ in either subtest, the higher persistence when the toy was unavailable does not necessarily support the hypothesis that terriers are more independent than herding dogs, but could also reflect higher motivation for the toy—although time interacting with the toy when it was available in the ‘food in box’ subtest did not differ between the breed groups.

## Conclusions

The findings of the current study give evidence that dogs’ human-directed gazing serves a communicative function. First, as long as an alternative reward was available, there was a positive correlation between interaction with the box (i.e., persistence) and gazing at people, suggesting that both behaviours served to obtain the inaccessible reward. Our study design thus allowed to some extent to tease apart gazing in relation to the unsolvable problem and for other reasons. While not all gazing is help-seeking, some gazing meets the requirements to be interpreted as such.

Second, dogs clearly differentiate between the two people present, gazing preferentially at the owner when faced with an unsolvable problem (even though significantly less so when the experimenter has handled the rewards), but gazing longer at whoever last handled the rewards in relation to toy play. This demonstrates that dogs are not looking randomly at visually salient objects in the environment. Moreover, the fact that within the same subtest, dogs in the experimenter-responsible group looked longer at the owner in relation to the box but longer at the experimenter in relation to the available toy rules out that one of the people was for some reason more visually salient than the other. These differential findings indicate that neither situational associations of a person with the reward nor the lifetime reinforcement history with the owner can fully explain gazing behaviour during the experiment. Likely, it is influenced by a combination of both, as well as by the dog’s current goal (here: obtaining the inaccessible reward vs playing).

Third, dogs demonstrated gaze alternations—a hallmark of communicative intent—and, like absolute duration of gazing, they adjusted the direction of gaze alternations depending on whether the owner or the experimenter was responsible for handling the reward.

To conclude, we show evidence that ‘looking back’ constitutes a social problem-solving strategy in dogs, but this is not true for all human-directed gazing. Indeed, gazing at humans may also result from giving up, besides many other possible functions (such as in our study, possibly a play invitation). Dogs show clear differentiation in gazing behaviour towards the two people present, depending on (1) the people’s responsibility in handling the rewards, (2) the social relationship with the persons, and (3) the dog’s current goal.

## Supplementary Information

Below is the link to the electronic supplementary material.

Supplementary file1 (PDF 110 KB)

Supplementary file2 (XLS 148 KB)

## Data Availability

The dataset generated during the current study is provided as supplementary information (Supplementary Information 2).
