# Supplementary material for: Disentangling help-seeking and giving up: differential human-directed gazing by dogs in a modified unsolvable task paradigm
Source: Anim Cogn. 2022 Jan 12;25(4):821–36. doi: 10.1007/s10071-021-01595-0 (PMC8753593; doi:10.1007/s10071-021-01595-0)
Supplement: Supplementary file 1 — Supplementary file1 (PDF 110 KB) [file 10071_2021_1595_MOESM1_ESM.pdf]

Disentangling help-seeking and giving up: differential human-directed gazing by dogs in a modified unsolvable task paradigm

Annina Hirschi, Alja Mazzini, Stefanie Riemer (https://orcid.org/0000-0001-8008-5291)

Companion Animal Behaviour Group, Division of Animal Welfare, Vetsuisse Faculty, University of Bern, 3012 Bern, Switzerland

Corresponding author: riemer.stefanie@gmail.com

Electronic Supplementary Material 1

Table S1. Demographic data of subjects, designation to the owner-responsible or the experimenter-responsible group, list of selected dog sports and, separately, whether the dogs participated in agility or search and rescue. For sports, obedience, rally obedience and international companion dog exams were subsumed as “Obedience”. Agility and Hoopers agility were subsumed as “Agility”. Search and rescue, nosework, and (man)trailing were subsumed as “Nosework” in the last column

ID	Breed group	Breed	Age (years)	Sex/ neuter status	Responsible person	Dog sports	Agility	Nose work
1	Herding	Australian shepherd	2.36	Female neutered	Owner	None indicated		
2	Herding	Border collie X Bergamasker	1.41	Female intact	Owner	Obedience, dogdance, nosework		1
4	Herding	Beauceron	5.84	Female intact	Owner	Obedience, nosework		1
7	Herding	Border collie	9.09	Male neutered	Owner	Obedience, nosework		1

8	Herding	Working kelpie	2.75	Female neutered	Owner	Obedience, agility, canicross, herding	1	
10	Herding	German shepherd	2.88	Female intact	Owner	Agility	1	
14	Herding	German shepherd	6.13	Female intact	Owner	Agility, dogdance, nosework, dummy training, frisbee	1	1
20	Herding	Bearded collie	6.38	Male neutered	Owner	Agility, dogdance, nosework	1	1
25	Herding	Beauceron	7.53	Female intact	Owner	Nosework, search and rescue		1
26	Herding	Pyrenean shepherd	7.51	Female intact	Owner	Obedience, nosework, Schutzhund		1
30	Herding	Border collie	2.93	Male neutered	Owner	Obedience, agility, nosework	1	1
32	Herding	Tervueren	2.32	Female neutered	Owner	Agility	1	
42	Herding	Australian shepherd	1.84	Female neutered	Owner	Nosework, dummy training, former search and rescue dog		1
51	Herding	Bearded collie X Border collie	6.15	Female neutered	Owner	Nosework, Schutzhund		1

3	Terrier	Border terrier	3.14	Female intact	Owner	Agility	1	
11	Terrier	Jack Russel terrier	8.18	Female neutered	Owner	None indicated		
13	Terrier	Irish terrier	8.61	Male neutered	Owner	NA	NA	NA
18	Terrier	Fox terrier	7.33	Male neutered	Owner	None indicated		
24	Terrier	Cairn terrier	6.69	Female neutered	Owner	Dogdance, search and rescue, herding		1
27	Terrier	Jack Russel terrier	2.56	Female neutered	Owner	Obedience, agility, trick training	1	
28	Terrier	Patterdale terrier	9.2	Female neutered	Owner	Agility, dogdance, nosework	1	1
36	Terrier	Border terrier	2.59	Female intact	Owner	None indicated		
38	Terrier	Border terrier	8.43	Female intact	Owner	Canicross, herding		
39	Terrier	Airedale terrier	4.33	Male intact	Owner	NA	NA	NA
40	Terrier	Irish terrier	3.29	Female neutered	Owner	Obedience, nosework, search and rescue		1
43	Terrier	American Staffordshire terrier	2.59	Male neutered	Owner	Agility, herding	1	
54	Terrier	Border terrier	10.03	Male neutered	Owner	Obedience, agility, nosework	1	
5	Herding	Border collie	4.54	Male intact	Experimenter	Agility, dogdance,	1	1

						nosework, dummy training, frisbee		
6	Herding	Border collie	4.22	Male NA	Experimenter	Nosework, treibball		1
9	Herding	Border collie	8.75	Male intact	Experimenter	Agility, trailing, treibball	1	1
15	Herding	Border collie	2.91	Male neutered	Experimenter	Obedience, agility, nosework, search and rescue	1	1
19	Herding	Border collie	4.19	Male intact	Experimenter	Hoopers agility, agility, search and rescue	1	1
21	Herding	Border collie	2.39	Male intact	Experimenter	NA	NA	NA
23	Herding	Border collie	4.68	Male neutered	Experimenter	Agility, nosework	1	1
29	Herding	Border collie X Australian shepherd	7.75	Male neutered	Experimenter	None indicated		
31	Herding	Smooth collie	3.21	Male neutered	Experimenter	Nosework, lounging		1
35	Herding	Border collie	3.47	Female intact	Experimenter	Agility	1	
44	Herding	Border collie	3.27	Female intact	Experimenter	Obedience		
45	Herding	Australian shepherd	5.99	Male neutered	Experimenter	Obedience, nosework, Schutzhund		1

49	Herding	Australian Working Kelpie	9.62	Male neutered	Experimenter	Nosework		1
52	Herding	Border collie	8.77	Female neutered	Experimenter	Agility, dogdance, nosework	1	
56	Herding	Border collie	7	Female intact	Experimenter	None indicated		
12	Terrier	Parson Russell terrier	6.7	Male neutered	Experimenter	Obedience, nosework, search and rescue		1
16	Terrier	Bull terrier	6.33	Female neutered	Experimenter	Nosework		1
17	Terrier	Jack Russell terrier	7.02	Male neutered	Experimenter	NA		
22	Terrier	Scottish terrier	2.3	Female neutered	Experimenter	Obedience		
33	Terrier	Parson Russell terrier	3.41	Female intact	Experimenter	Obedience, dogdance		
34	Terrier	Jack Russell terrier	1.8	Female intact	Experimenter	None indicated		
37	Terrier	Parson Russell terrier	10.34	Male neutered	Experimenter	Obedience, agility, nosework	1	1
41	Terrier	Scottish terrier	1.3	Male intact	Experimenter	Agility, flyball	1	
46	Terrier	Border terrier	7.61	Male intact	Experimenter	Obedience, agility, dogdance, nosework, dummy	1	1

						training, canicross		
47	Terrier	Border terrier	5.41	Male intact	Experimenter	Obedience, agility, canicross, herding	1	
48	Terrier	Jack Russell terrier	8.25	Female neutered	Experimenter	NA	NA	NA
50	Terrier	Parson Russell terrier	10.15	Female neutered	Experimenter	Agility, nosework	1	1
53	Terrier	Jack Russell terrier	1.88	Male neutered	Experimenter	None indicated		
55	Terrier	Jack Russell terrier	8.92	Male neutered	Experimenter	Herding		

Table S2. Intra-class correlation coefficients (two-way, random, consistency, single measures) for durations

Variable	ICC
Toy in box – Duration of gazing directed at the owner	0.986
Toy in box – Duration of gazing directed at the experimenter	0.984
Food in box – Duration of gazing directed at the owner	0.984
Food in box – Duration of gazing directed at the experimenter	0.979
Toy in box – Duration of interacting with the box	0.994
Food in box – Duration of interacting with the box	0.999
Food in box – Duration of interacting with the toy	1.0
Toy in box – Duration of interacting with the food (puzzle)	0.997

Table S3. Intra-class correlation coefficients (two-way, random, absolute consistency, single measures) for frequencies

Variable	ICC
Frequency of gaze alternations box-owner	0.953
Frequency of gaze alternations owner-box	0.969
Frequency of gaze alternations box-experimenter	0.888
Frequency of gaze alternations experimenter-box	0.834

Table S4. Results of Mann Whitney U tests testing for differences between the experimenter-responsible and the owner-responsible group in persistence during both subtests, time interacting with the food during the ‘toy in box’ subtest, and time interacting with the toy during the ‘food in box’ subtest

Dependent variable	*U*	*p*
Toy in box – total time interacting with box	341	0.408
Food in box – Total time interacting with box	333.5	0.342
Toy in box – total time interacting with food (puzzle)	353	0.528
Food in box – total time interacting with toy	351	0.507

Table S5. Median durations and (in parentheses) inter-quartile range of interaction with the box in both subtests and interaction with the food during the ‘toy in box’ subtest and the toy during the ‘food in box’ subtest in the experimenter-responsible (Group E) and the owner-responsible group (Group O)

Dependent variable	Group E	Group O
Toy in box – total time interacting with box	33.8 (15.4; 46.6)	27.6 (12.2; 46.8)
Food in box – Total time interacting with box	29.2 (7.4; 41.2)	13.6 (0; 39.8)
Toy in box – total time interacting with food (puzzle)	35.30 (24.2; 51.3)	39.0 (17.6; 43.4)

Food in box – total time interacting with toy	47.6 (4.4; 130.1)	68 (6.0;115.2)

Table S6. Results of Mann Whitney U tests assessing the effect of mask wearing on human-directed gazing in the experimenter-responsible group. Seventeen dogs were tested without a mask, and 12 dogs were tested with a mask.

**Dependent variable**	***U***	***p***
Toy in box – total time looking at owner	94	0.72
Toy in box – total time looking at experimenter	82.5	0.39
Toy in box – proportion of looking at the owner	87	0.51
Food in box – proportion of looking at the owner	67	0.12
Food in box – total time of box-related looking at the owner	90	0.60
Food in box – total time of box-related looking at the experimenter	92	0.66
Food in box – total time of toy-related looking at the owner	89	0.56
Food in box – total time of toy-related looking at the experimenter	81	0.35

Fig. S1. Boxplot of the duration of interaction with the box during the 'food in box' subtest, indicating that the three highest durations of interaction with the box are outliers

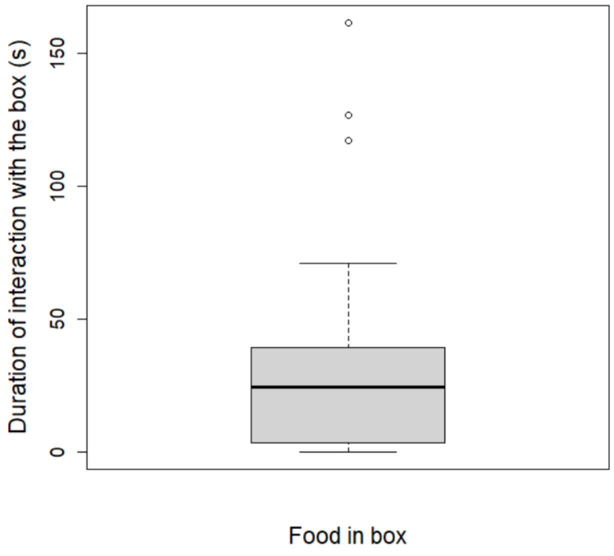

Table S7. Sequential Bonferroni correction was applied separately for each of the six families (Voelkl 2019) of tests (1. correlations of persistence with gazing; 2. group differences in latency to gaze at the owner vs the experimenter, 3. within-group differences in latency to gaze at the owner vs the experimenter, 4. group differences in the proportion of gazing at the owner; 5. group differences in gaze alternations; 6. differences between the breed groups). Presented are the test statistics, original p-values, corrected alpha levels after sequential Bonferroni correction, and whether results are considered significant after correction for each family. Results that remained significant after correction are marked by an asterisk in the "Significance after correction" column. Results that are no longer significant after correction are identified as "n.s. after correction". Results that never achieved p values <0.05 before Bonferroni correction are identified as "n.s."

**Correlations**	r_S	p	**Bonferroni-corrected α**	**Significance after correction**
Food in box – duration of interaction with the box ~ duration of box-related gazing at people	0.55	0.00001	0.01	*
Toy in box (until food consumed) – duration of interaction with the box ~ duration of gazing at people	0.51	0.00005	0.0125	*
Toy in box (total time) – duration of interaction with the box ~ total duration of gazing at people	-0.42	0.00136	0.0167	*
Food in box – duration of interaction with the box ~ total duration of gazing at people	0.29	0.029	0.025	n.s. after correction
Food in box – duration of interaction with the box ~ duration of toy-related gazing at people	-0.28	0.0336	0.05	*
**Difference between Group O and Group E in the latency to first gaze at the owner and the experimenter**	U	p	**Bonferroni-corrected α**	**Significance after correction**
Toy in box - Latency to first gaze at the experimenter	146.0	<0.0001	0.0125	*
Food in box - Latency to first gaze at the owner	149.0	0.146	0.0167	n.s.
Toy in box - Latency to first gaze at the owner	293.5	0.159	0.025	n.s.
Food in box - Latency to first gaze at the exoerimenter	172.0	0.397	0.05	n.s.

**Difference within groups to first gaze at the owner vs the experimenter**	***Z***	***p***	**Bonferroni-corrected α**	**Significance after correction**
Group O/ Toy in box - Latency to gaze at the owner vs the experimenter	2.96	0.003	0.0125	*
Group O/ Food in box - Latency to gaze at the owner vs the experimenter	2.34	0.019	0.0167	n.s. after correction
Group E/ Food in box - Latency to gaze at the owner vs the experimenter	0.31	0.753	0.025	n.s.
Group E/ Toy in box - Latency to gaze at the owner vs the experimenter	0.23	0.820	0.05	n.s.
**Difference between Group O and Group E in the proportion of gazing at the owner**	***U***	***p***	**Bonferroni-corrected α**	**Significance after correction**
Food in box – Proportion of total gaze duration directed at the owner	90	0.00001	0.0125	*
Toy in box – Proportion of total gaze duration directed at the owner	143	0.00005	0.0167	*
Food in box – Proportion of total toy-related gaze duration directed at the owner	67.5	0.00097	0.025	*
Food in box – Proportion of total box-related gaze duration directed at the owner	95.5	0.035	0.05	*
**Difference between Group O and Group E in the frequency of gaze alternations**	***U***	***p***	**Bonferroni-corrected α**	**Significance after correction**
Toy in box – Number of gaze alternations involving the experimenter	134	0.00002	0.0125	*
Food in box – Number of gaze alternations involving the experimenter	208.5	0.0027	0.0167	*
Toy in box – Number of gaze alternations involving the owner	370.5	0.73	0.025	n.s.
Food in box – Number of gaze alternations involving the owner	387	0.94	0.05	n.s.
**Effect of breed group**	***U***	***p***	**Bonferroni-corrected α**	**Significance after correction**

Toy in box – Total time interacting with box	221	0.005	0.007	*
Toy in box – Total time gazing at a person (owner/experimenter)	304	0.151	0.0083	n.s.
Toy in box – Time interacting with the food (puzzle)	350	0.496	0.01	n.s.
Food in box – Total time gazing at a person (owner/experimenter)	371.5	0.743	0.0125	n.s.
Food in box – Total time interacting with box	374.5	0.780	0.0167	n.s.
Food in box – Proportion of total gaze duration directed at the owner	314	0.850	0.025	n.s.
Toy in box – Proportion of total gaze duration directed at the owner	381	0.863	0.05	n.s.
